# Cluster of African Trypanosomiasis in Travelers to Tanzanian National Parks

**DOI:** 10.3201/eid0806.010432

**Published:** 2002-06

**Authors:** Tomas Jelinek, Zeno Bisoffi, Lucio Bonazzi, Pieter van Thiel, Ulf Bronner, Albie de Frey, Svein Gunnar Gundersen, Paul McWhinney, Diego Ripamont, for European Network on Imported Infectious Disease Surveillance

**Affiliations:** * Ludwig-Maximilians-University, Munich, Germany; † S. Cuore Hospital, Negrar, Verona, Italy; ‡Reggio Emilia Hospital, Reggio, Italy; § University of Amsterdam, Amsterdam, the Netherlands; ¶ Karolinska Hospital, Stockholm, Sweden; # Worldwide Travel Medical Consultants, Northcliff, South Africa; ** Ullevaal Hospital, Oslo, Norway; †† Bradford Royal Infirmary, Bradford, United Kingdom; ‡‡ General Hospital, Bergamo, Italy

**Keywords:** African trypanosomiasis, sleeping sickness, travel

## Abstract

Game parks in Tanzania have long been considered to be at low risk for African trypanosomiasis; however, nine cases of the disease associated with these parks were recently reported. The outbreak was detected through TropNetEurop, a sentinel surveillance network of clinical sites throughout Europe.

African trypanosomiasis (sleeping sickness), a serious infection caused by a protozoan (*Trypanosoma brucei*), is usually spread to humans by the tsetse fly via infected animals and humans. Although the World Health Organization has reported a dramatic increase in incidence in Africa, the disease has remained a rare but well-documented cause of fever in travelers returning from endemic areas. In recent years, infection in returning travelers has been more likely to be due to the East African form (caused by *T. brucei rhodesiense*), rather than the West African form (which is due to *T. brucei gambiense*); the latter form causes a fulminant illness for which rapid diagnosis is necessary (*1*,*2*). We report details of nine recent cases caused by the West African form of this disease, one fatal; all of the cases occurred in travelers to Tanzanian national parks.

## Case Reports

Game parks in Tanzania have long been considered to be low-risk areas for African trypanosomiasis (*3*). However, in February 2001, two index patients and seven additional European and South African patients were seen with trypanosomiasis acquired in the Tarangire and Serengeti National Parks, Tanzania (*4*). The patients were identified and reported in TropNetEurop, a sentinel surveillance network of clinical sites throughout Europe for monitoring imported infectious diseases.

All of the South African patients but one were European nationals (Table). To our knowledge, all patients had traveled to the Tarangire and Serengeti National Parks, in addition to a number of other destinations. This area in East Africa has been implicated as being endemic for African trypanosomiasis. However, the case incidence in Tanzanian and foreign nationals has been very low in recent decades.

**Table:** Patients with African trypanosomiasis, Tanzania

No.	Sex	Age	Nationality	Mo/yr of diagnosis	Clinical details and treatment	Travel history^a^
1	M	33	Italian	02/01	Skin lesion (back), fever, nausea/vomiting; no major complications; treatment with suramin	Tourist: Kenya; Lake Manyara, Serengeti, and Ngorongoro NPs
2	M	32	Italian	02/01	Skin lesion left leg; fever; multiorgan failure; anuria; treatment with pentamidine	Tourist: East Tsavo, Ngorongoro, and Serengeti NPs
3	F	44	British	02/01	Skin lesion left leg; fever; no major complications; treatment with suramin	Tourist: Nairobi, Amboseli, Lake Manyara, Ngorongoro, and Serengeti NPs
4	M	41	Swedish	03/01	Skin lesion right foot; fever; treatment with suramin	Tourist: Lake Manyara, Ngorongoro, Tarangire, and Serengeti NPs
5	M	68	South African	03/01	Fever; renal failure; acidosis; jaundice; DIC; treatment with melasoprol	Tourist: Serengeti NP
6	F	27	Norwegian	03/01	Skin lesion left side of face; fever; no complications; treatment with suramin	Research project on zebras: Ngorongoro and Serengeti NPs
7	M	60	Dutch	03/01	Fever; treatment with suramin	Tarangire NP
8	F	55	Dutch	04/01	Skin lesion left ankle; fever; headache; treatment with suramin	Tarangire NP
9	F	53	Dutch	06/01	Skin lesion right leg; fever; headache; intracerebral manifestation; coma; death; treatment with suramin and melasoprol	Lake Manyara, Ngorongo, and Serengeti NPs

^a^East Tsavo NP and Amboseli NP are in Kenya; all other NPs mentioned are in Tanzania. NP, national park; DIC, disseminated intravascular coagulopathy.

During their journey or briefly after their return, the patients, all febrile, were seen by general practitioners or emergency departments. Most patients were seen during the primary stage of disease (patients 1, 3, 4, 6, 7, 8; Table); however, several showed signs of the secondary stage, including cerebral manifestations. Most patients also showed a typical skin lesion, the trypanosome chancre. Diagnosis was established by thin and thick blood film. Although three patients had multiorgan failure, and specific medication was difficult to obtain, drug treatment proved successful in all but one patient, who died. Drugs for treatment were not chosen for the clinical stage the patients exhibited but rather for availability. Thus, patients with complications and a manifest secondary stage of disease received pentamidine only.

## Conclusions

The temporal clustering of imported cases suggests a change in the local epidemiology of this disease and may herald further cases in tourists during the current travel season. For 1998, the World Tourism Organization recorded 450,000 visitors to Tanzania (*5*), for a potential annual incidence of trypanosomiasis in tourists to Tanzania of at least 9/450,000. This is an increase from near zero during recent years to 2/100,000. The risk for those visiting the Tarangire and Serengeti National Parks is obviously higher. Reaction of the Tanzanian authorities involved strengthening installation of insecticide-impregnated locations in Serengeti to include roads, lodges, staff quarters, and campsites. This initial program resulted in a dramatic decline of tsetse flies in Serengeti during the second half of 2001. This effort will have to be sustained by mandatory killing of flies at some keys areas including Serengeti, Tarangire, and Lake Manyara National Parks. The National Medical Research Program has been directed to screen more people for the disease around these foci.

For many of the patients, drugs for treatment were extremely difficult to obtain. For some European patients, treatment with suramin was possible only after informal help from member sites of the network. Drugs for treatment (suramin, melasoprol, and eflornithin) have now been obtained. Surveillance in cattle to establish their role in the epidemiology of the disease will also be conducted (Tanzania Chief Veterinary Officer, pers. comm.).

This report highlights the effectiveness and importance of sentinel surveillance methods for monitoring imported infectious diseases in Europe. TropNetEurop, the network that identified and reported the index cases, is known for its speed of reporting, often within days of diagnosis. The network’s use of member sites as regional referral centers is based on an anonymous reporting system at sentinel clinics. Discussion of the index patients by member sites triggered increased awareness within the network and led to the rapid recording of additional patients and a pattern that might have otherwise gone undetected.
